# miR-204-5p Suppress Lymph Node Metastasis via Regulating CXCL12 and CXCR4 in Gastric Cancer

**DOI:** 10.7150/jca.33273

**Published:** 2020-03-05

**Authors:** Juan Zhang, Ling Xing, Hongwei Xu, Kaixuan Wang, Junjun She, Feiyu Shi, Hongyu Wu, Yongjie Sun, Jun Gao, Shuixiang He

**Affiliations:** 1 Department of Gastroenterology, The First Affiliated Hospital of Xi'an JiaoTong University, 277 Yanta West Road, Xi'an, Shaanxi, China.; 2 Department of Gastroenterology, Eastern Hepatobiliary Hospital, Second Military Medical University, Shanghai, China.; 3 Department of Gastroenterology, Changhai Hospital, Second Military Medical University, Shanghai, China.; 4 Department of Gastroenterology, Kunshan traditional Chinese medicine hospital. Jiangsu, China.; 5 Department of General Surgery, The First Affiliated Hospital of Xi'an Jiao Tong University, 277 Yanta West Road, Xi'an, Shaanxi, China.; 6 Institute of Oncology, Second Affiliated Hospital, Xi'an Medical College, China.

**Keywords:** miR-204-5p, CXCR4, CXCL12, gastric cancer, migration, invasion, lymph node metastasis

## Abstract

Gastric cancer (GC) exhibits a poor prognosis due to extensive invasion and lymphatic metastasis in the advanced stage. In this study, we firstly found that the expression of miR-204-5p markedly decreased in GC patients' tissue and serum, especially in GC with lymphatic metastasis. And ROC analysis showed miR-204-5p also served as a predicted factor for the lymphatic metastasis of GC. CXCL12 and CXCR4 were predicted and confirmed as the functional targets of miR-204-5p by Targetscan analysis, dual luciferase assay and western blotting analysis. In addition, we further determined that miR-204-5p suppresses migration and invasion in GC. This finding elucidates new functions and mechanisms for miR-204-5p in GC development and provides a new potential diagnostic marker and therapeutic targets for GC.

## Introduction

Gastric cancer (GC) is a major public health problem, ranking the fourth most frequently diagnosed cancer and the second in cancer-associated death worldwide 1. Although the diagnosis and treatment of GC have been improved, the 5-year overall survival rate of GC remains poor, largely due to diagnostics in advanced stages 2,3. Extensive invasion and lymphatic metastasis are the main biological characteristics factors responsible for the poor prognosis of advanced stages of GC patients 4,5. Therefore, elucidation of the mechanisms underlying GC invasion and metastasis will provide essential clues to understand GC pathogenesis.

Currently, accumulating evidence demonstrated that microRNAs (miRNAs), as a class of endogenous noncoding short RNA serve as oncogenes and onco-suppressors in diverse human carcinogenesis including GC 6-9. Many of them are associated with crucial biological processes, such as cell proliferation, metastasis, and invasion, by regulating relevant targets gene expression at post-transcriptional levels. For example, miR-589 markedly promotes GC metastasis and invasion via an atypical miR-589-LIFR-PI3K/AKT-c-Jun feedback loop 10. miRNA-21 promotes the growth of gastric cancer cells by adjusting and controlling PEG2 11. Similarity, previous expression profiling data uncovered that as an onco-suppressors, miR-204-5p significantly downregulated in GC tissue 12-14. It plays an important role in regulating metastasis, promoting cell apoptosis, and inhibiting cell proliferation 15-17. However, the potential role for miR-204-5p in lymphatic metastasis of GC has yet to be examined. Therefore, in the present study, the molecular mechanism underlying miRNAs regulating lymphatic metastasis of GC was investigated.

## Methods

### Clinical specimens

The 86 GC patients and 72 benign patients were recruited. The data of the patients were shown in Table 1. Among the 86 CG patients, the 44 pairs of both the tissues and its matched tumor-adjacent gastric tissue were recruited. The histopathology diagnosis was based on the surgical gastric resection tissues and biopsy specimens in the First Affiliated Hospital of Xi'an Jiaotong University. This study protocol was approved by the Institutional Review Board and Ethics Committee of the First Affiliated Hospital of Xi'an Jiaotong University.

### Microarray analysis

Serum from GC (n=4) and benign (n=4) patients was used to construct miRNA microarray. Total RNA was extracted from the 400 µl serum using the mirVana Total RNA Isolation kit (Thermo Fisher). The miRNA gene expression microarray analysis was performed using Agilent Human miRNA Microarray, Release 21.0, 8x60K (Oebiotech, Shanghai, China).

### RNA isolation and Quantitative PCR analysis

The detection of miR-204-5p expression levels in the plasma samples referred to the previously established method 18. Briefly, all plasma samples were thawed on ice and 200 µl of each sample was transferred to a tube containing 750 µl TRI Reagent BD (Molecular Research Center, Inc., Cincinnati, USA) and 20 µl 5 mol/l acetic acid. Five microliters of synthetic C. elegans miRNA cel-miR-39 (50 pmol/l, synthetic RNA oligonucleotides synthesized by Qiagen) was spiked into each sample as a control after initial plasma denaturation for RNA isolation. Each obtained total RNA pellet was re-suspended in 40 µl of nuclease-free water and stored at -80 °C. Then a 2 µl aliquot was taken from the 40 µl solution of re-suspended total RNA (equivalently to the RNA extracted from 10 µl of plasma) was reverse transcribed by TaqMan MicroRNA Reverse Transcription Kit (ThermoFisher). Then, 2 µl of the cDNA solution was amplified in a final volume of 20 µl using TaqMan Universal Master Mix (ThermoFisher). Levels of mature miR-204-5p (Assay ID 000508) were measured using TaqMan MicroRNA Assay (ThermoFisher) by normalizing to the levels of control cel-miR-39(Assay ID 000200).

For the detection of miR-204-5p expression levels in the tissues, total RNA was extracted using the mirVana miRNA isolation kit (Ambion). Levels of mature miR-204-5p (Assay ID 000508) were measured using TaqMan MicroRNA Assay (ThermoFisher) by normalizing to the levels of control U6 (Assay ID 001973).

### Cell culture and treatment

The human GC cell lines SGC7901, MGC803 were purchased from the Cell Bank of the Chinese Academy of Sciences. Cells were incubated at 37 °C in a humidified atmosphere containing 5% CO2 and cultured in DMEM (Gibco, ThermoFisher Scientific, Inc., Grand Island, NY, USA) combined with 10% fetal bovine serum (FBS). miR-204-5p mimics were purchased from QIAGEN. For transient transfection of cells in six-well plates, 100 uM mimics was added with lip2000 in OPTI-MEM media (Gibco; ThermoFisher Scientific, Inc) according to the manufacturer's instructions.

### Dual-luciferase reporter assay

The effects of miR-204-5p overexpression on the CXCR4 and CXCL12 activity were measured by dual-luciferase reporter assays. The plasmids of the wild-type (3'UTR-W) and corresponding mutant-type (3'UTR-M) containing CXCR4 and CXCL12 3'UTR cDNA were constructed based on the pGL3 vector (Promega Corp., Madison, WI, USA; GenBank® Accession Number U47296, Catalog number selected: E1741). The 293T cells were con-transfected with the miR-204-5p mimics or negative control (Nc) and the plasmid 3'UTR-W and 3'UTR-M of CXCR4 or CXCL12. After 24 h, the dual-luciferase assays were performed using the dualluciferase reporter assay system (Promega, Madison, WI, USA) with a Victor X machine (PerkinElmer, Norwalk, CT, USA).

### Western blotting

The whole cell protein was extracted using RIPA buffer combined with protease inhibitor (Merck Millipore). The protein concentration was determined by the Bradford method (Beyotime, Shanghai, China). Protein was separated by 10% SDS/PAGE (Beyotime) and transferred to polyvinylidene fluoride membrane (Merck Millipore). The members were blocked in 5% skim milk with TBST for 1 h at room temperature. Then, the primary antibodies were incubated at 4 °C overnight and secondary antibodies for 1 h at room temperature. The primary antibodies used were mouse anti-GAPDH (Sigma, USA), rabbit anti-CXCR4 and rabbit anti-CXCR12 (Proteintech, Rosemont, IL, USA).

### Migration and invasion assay

Migration and invasion were performed to determine the migratory ability and invasiveness of enforced expression of miR‑204‑5p. For the cell scratch-wound assays, cells were grown in six-well plates until confluent. A wound was generated on the surface of the resulting cell monolayer via scraping with the 10-uL tip of a pipette. After that, the cells were incubated for 12-48 hours. The cells in the wounded monolayer were photographed at different time points, and cell migration was assessed by measuring gap sizes in multiple fields. For the Boyden chamber assay, 24-well tissue culture plates with 12 cell culture inserts (Millipore) were used. Each insert contained an 8-um-pore-size polycarbonate membrane with a pre-coated thin layer of a basement membrane matrix (ECMatrix for the invasion assay). Ten percent fetal bovine serum-containing medium was placed in the lower chambers to act as a chemoattractant. Cells (5×104) in a 300-uL volume of serum-free medium were placed in the upper chambers and incubated at 37 °C for 48 hours. Cells on the lower surface of the polycarbonate membrane were stained, counted, and photographed under a microscope.

### Statistical analysis

Statistical analysis was performed using the SPSS version 17.0 software package (IBM Corp., Armonk, NY, USA). Each experiment was repeated at least three times. Independent sample Student's t-test was used if the quantitative data between groups show normal distribution. If not consistent with the normal distribution, uses the Wilcoxon-Mann-Whitney test. A two-tailed χ^2^ test or the Fisher exact test was performed to determine the significance of enumeration data. A p-value ≤0.05 was considered statistically difference.

## Results

### Identification of differentially expressed miRNAs profiles in GC

**Figure 1:**
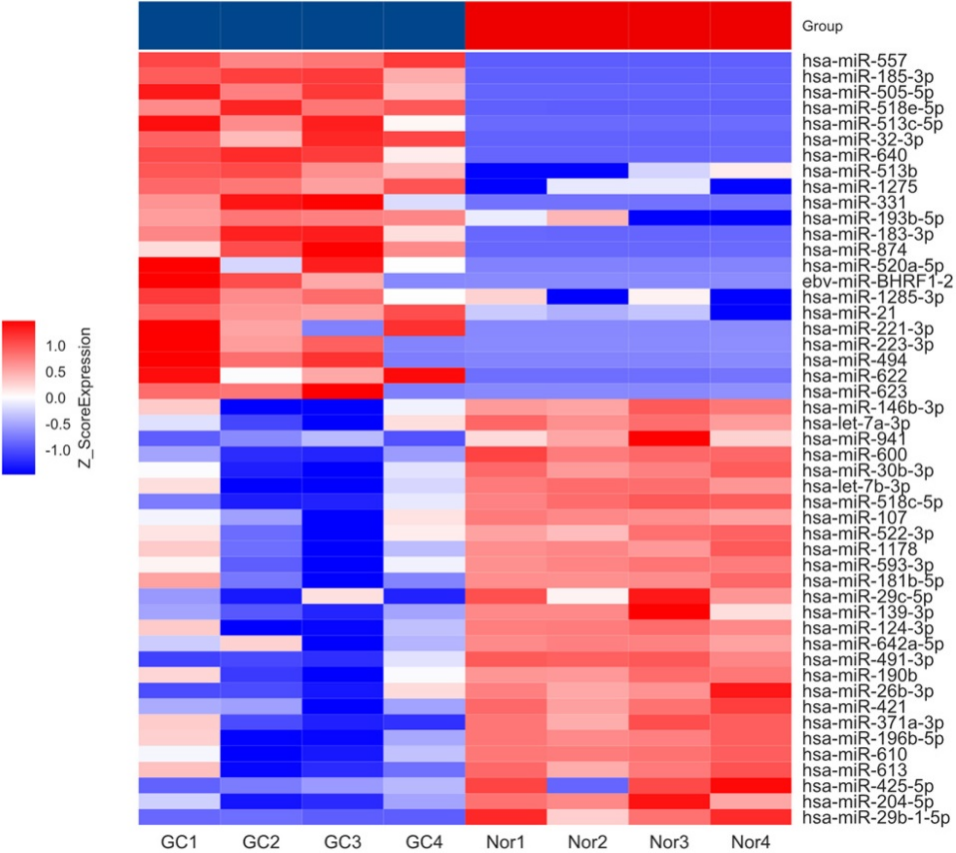
** Heatmap showing differential miRNA expression in GC and benign patients' serum.** GC: gastric cancer; Nor: benign patients.

To investigate the expression pattern of miRNAs in GC patient's, serum from GC (n=4) and benign (n=4) patients were used to construct miRNA microarray. The miRNA microarray results showed significantly differential miRNA expression profiles between serum from GC and benign patients (fold change ≥ 1.5, P < 0.05). The heat map presented in Figure 1. And we found that miR-204-5p significantly down-regulated in GC patient's serum compared with benign patients'.

### miR-204-5p downregulated in GC patients serum and tissues

**Figure 2:**
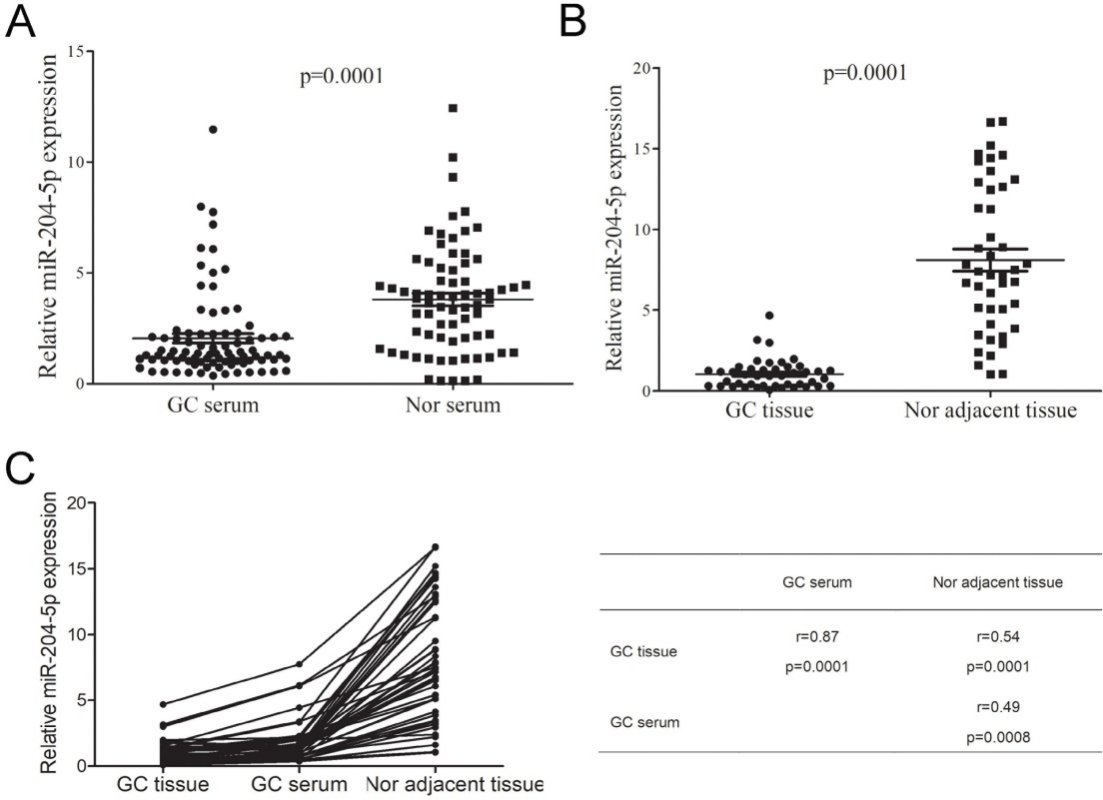
** miR-204-5p expression in GC patients serum and tissues. A,** miR-204-5p expression in GC and benign patients' serum. **B,** miR-204-5p expression in GC tissues and its corresponding tumor-adjacent gastric tissue. **C,** Correlation between GC tissue, serum and tumor-adjacent gastric tissue. GC, gastric cancer. Nor, benign patients.

To further determine the expression level of miR204-5p in GC, we firstly investigated miR-204-5p expression in 86 GC and 72 benign patients' serum using RT-qPCR. As shown in Figure 2A, we observed that the miR-204-5p expression in GC patients' serum markedly decreased. Furthermore, the miR-204-5p expressions in 44 GC tissues and its compared tumor-adjacent gastric tissue were also investigated. The RT-qPCR analysis shown miR-204-5p levels significantly decreased in GC tissues compared to adjacent non-tumor gastric tissue (Figure 2B). In addition, we found that there were significant correlation between GC tissue, serum and tumor-adjacent gastric tissue (Figure 2C). These findings indicated that the down-regulation of miR-204-5p could contribute to GC development and progression.

### miR-204-5p has a negative effect on lymph node metastasis in patients with GC

**Figure 3:**
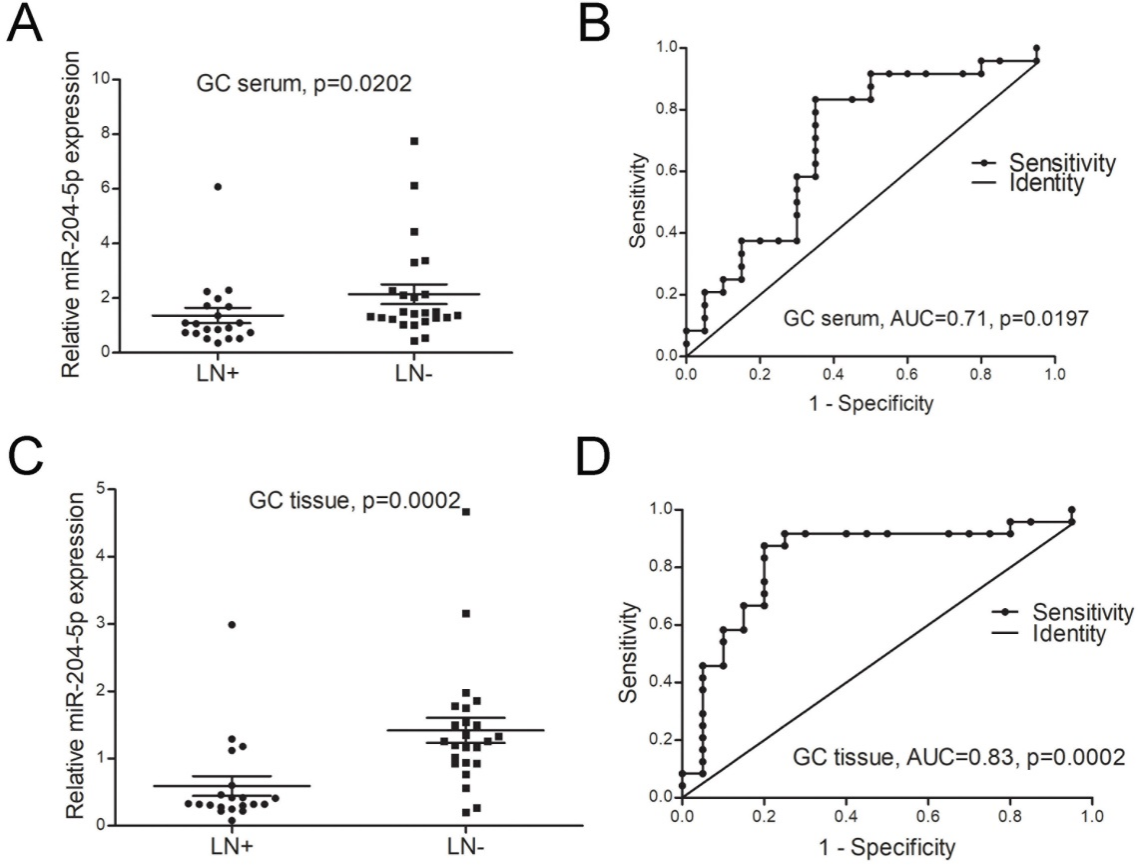
** miR-204-5p is a negative effect on the lymph node metastasis of patients with GC. A,** the expression level of miR-204-5p in GC patients' serum with LN metastasis and without LN metastasis. **B,** the efficacies of miR-204-5p in GC patients' serum distinguished LN metastasis from without LN metastasis patients. **C,** the expression level of miR-204-5p in GC patients' tumor tissues with LN metastasis and without LN metastasis. **D,** the efficacies of miR-204-5p in GC patients' tissues distinguish LN metastasis from without LN metastasis patients. LN+, GC with LN metastasis. LN-, GC without LN metastasis.

The relationship between miR-204-5p expression and clinicopathological characteristics was investigated in this study. As shown in Table 2 and Figure 3A&B, the expression of miR-204-5p both in GC patients' serum and tumor tissues were significantly associated with lymph node (LN) metastasis and clinical stage. The ROC analysis showed the expression of miR-204-5p both in serum and tissue can be used to distinguish LN metastasis from without LN metastasis patients (AUC for serum=0.71, AUC for tissue=0.83, Figure 3C, D). These results indicated that miR-204-5p was a negative effect on the lymph node metastasis of patients with GC.

### miR-204-5p targets the 3'-UTR of the both CXCR4 and CXCL12

**Figure 4:**
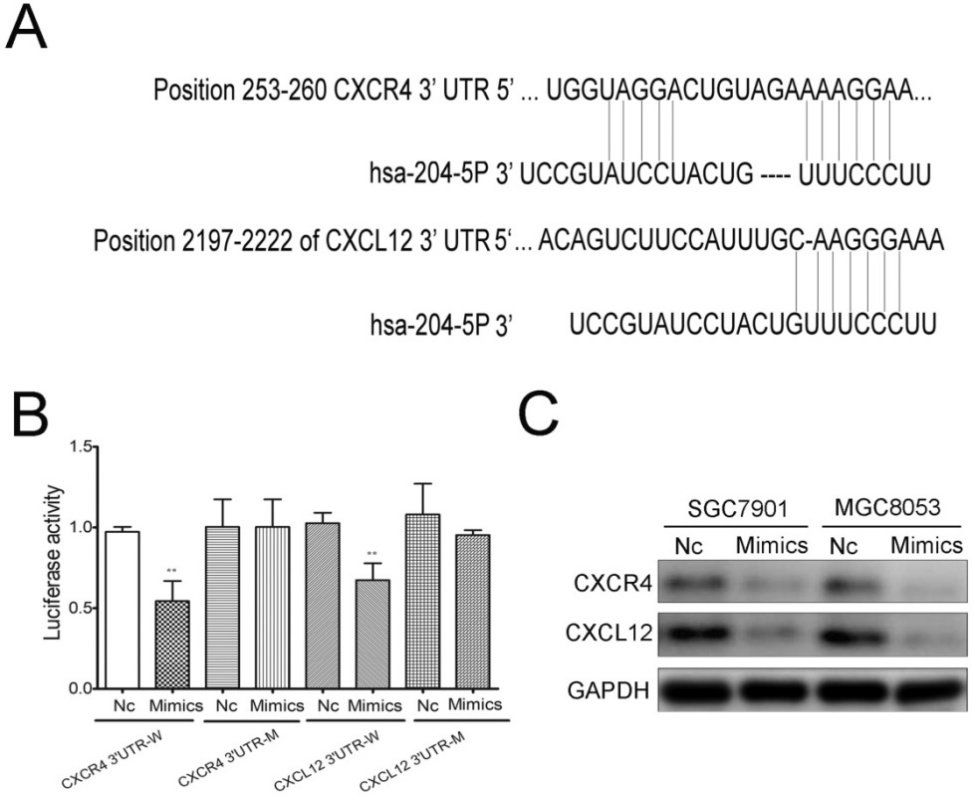
** CXCR4 and CXCL12 are the targets of miR-204-5p. A,** The binding sites of mir-204-5p within the 3'-UTR of CXCR4 and CXCL12 were predicted by Targetscan. **B,** Mimics of miR-204-5p suppressed luciferase activity in cells with the wild-type plasmid of CXCR4 and CXCL12 3'UTR-W, but did not cause a significant change in cells with their corresponding mutant-type plasmid 3'UTR-M. C, Western blots showed the change of CXCR4 and CXCL12 protein expression after the transfection with a miR-204-5p mimic or negative control. NC: negative control ,** :p<0.01.

To further identify the mechanism of miR-204-5p in GC cells, Targetscan (http://www.targetscan.org/) was used to investigate the direct targets of miR-204-5p. The binding sites of miR-204-5p matched the 3'-UTR of CXCR4 and CXCL12, suggesting that CXCR4 and CXCL12 are potential targets of miR-204-5p (Figure 4A). To further determine whether miR-204-5p could directly bind to the 3'-UTR of CXCR4 and CXCL12, a dual luciferase reporter assay was employed. Luciferase reporter plasmids containing the wild-type and mutant binding sites were constructed and transfected in 293 cells. The results suggested that miR-204-5p could suppress reporter gene activity of the wild-type 3'-UTR but not the mutant type, which indicated that CXCR4 and CXCL12 were the direct targets of miR-204-5p (Figure 4B). Furthermore, we transfected SGC7901 and MGC803 cells with the miR-204-5p mimics, and western blotting analysis showed that enforced expression of miR-204-5p led to a markedly decrease in the expression of CXCR4 and CXCL12 (Figure 4C). Overall, we demonstrated that CXCR4 and CXCL12 are the functional targets of miR-204-5p in GC.

### miR-204-5p suppresses GC cell migration and invasion

**Figure 5:**
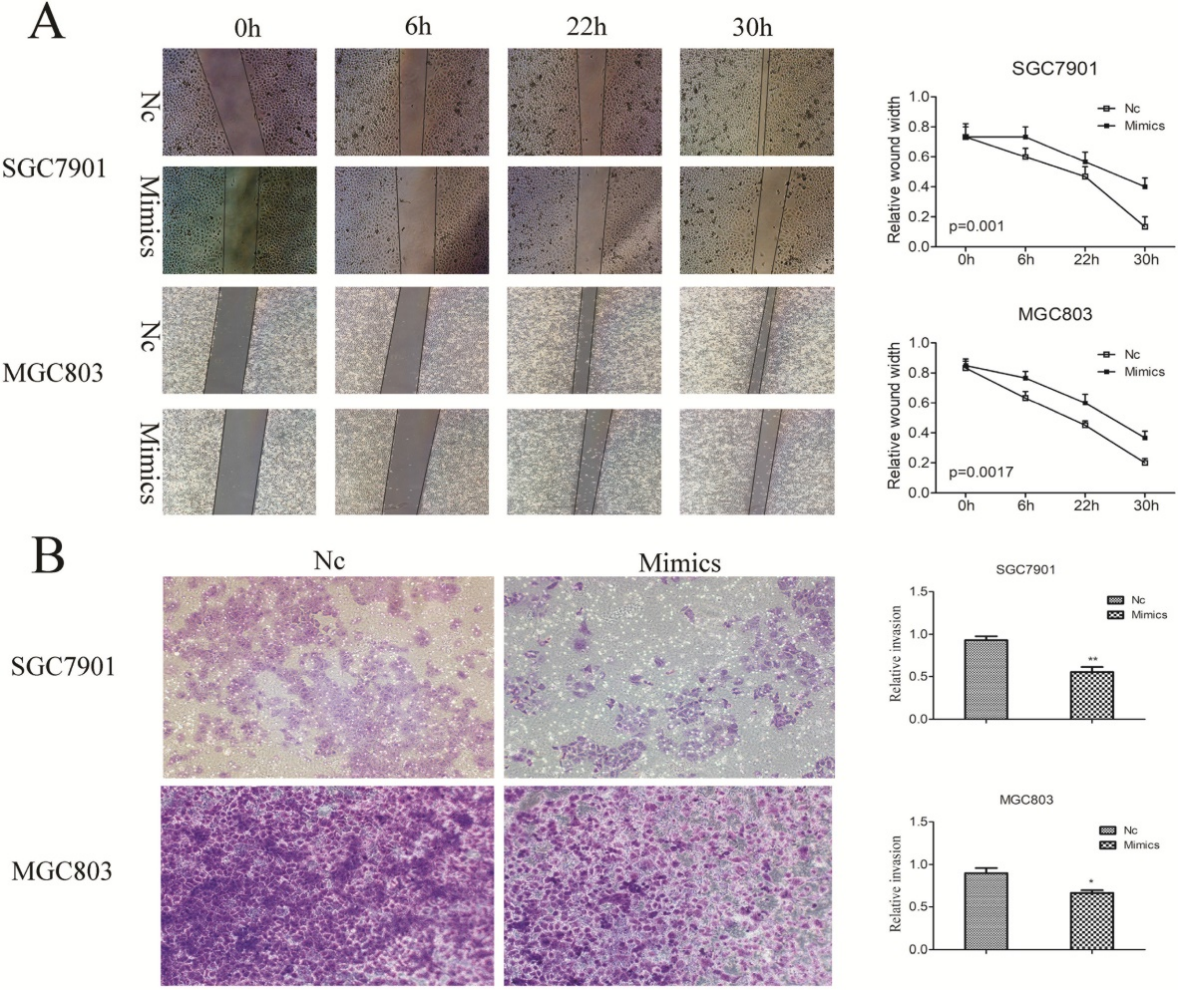
** Influence of enforced miR-204-5p expression on GC cell migration and invasion. A,** miR-204-5p suppresses GC cell migration. **B,** miR-204-5p suppresses GC cell invasion.

To determine the effect of the miR-204-5p expression on GC migration and invasion, miR-204-5p mimics were transfected in SGC7901 and MGC803 cells. We had wounded the transfected cells via scratching and maintained them for at least 48 hours. The results demonstrated that enforced expression of miR-204-5p strongly inhibited the flattening and spreading of SGC7901 and MGC803 cells (Figure 5A). Similarly, transwell assays revealed that the invasiveness of miR-204-5p mimics-transfected cells were much lower than those of control cells (Figure 5B). Taken together, these results further confirmed the tumor suppressor role of miR-204-5p in metastasis of GC.

## Discussion

Metastasis is a critical factor contributed to cancer mortality for patients at the advanced stage 19. In GC patients, amounting reports have revealed that LN is the main issue of metastatic destinations and patients with LN metastasis means the poor outcome 20,21.Therefore, inhibition of LN metastasis could be an effective therapeutic approach to improve GC patient's prognosis. In this study, we report that miR-204-5p expression markedly decreased GC patient's serum and tissue. The thorough analysis indicates that lower expression of miR-204-5p was positively associated with LN metastasis. Then, the underlying molecular mechanism of miR-204-5p in GC was further investigated. We discovered that both CXCR4 and CXCL12 genes were the direct targets of miR-204-5p. Furthermore, Vitro biological function experiments also revealed that miR-204-5p had a potent tumor suppressor role in migration and invasion inhibition. All of these findings were strongly indicate that miR-204-5 act as a metastasis suppressor in GC and could be a potential therapeutic target for the treatment of GC.

Recent extensive studies have demonstrated that miR-204-5p exerts antitumor effects by controlling cell proliferation, apoptosis, metastasis, invasion, angiogenesis and chemotherapeutic sensitivity 22-26. Zhang, B et al. study uncovered that miR-204-5p expression was negatively related to the tumor TNM stage in GC 17. However, the molecular mechanisms underlying miR-204-5p controlling LN metastasis have been seldom studied. In present study, we examined the expression of miR-204-5p in GC tissue, tumor-adjacent gastric tissue and serum, and found that miR-204-5p expression markedly decreased in GC tissue and serum. In addition, our current study for the first time established that the frequent lower expression of miR-204-5p correlated with LN metastasis. And the ROC curve revealed that lower expression of miR-204-5p may serve as a predicted factor for LN metastasis in GC patients. These findings underscored a potentially important role of miR-204-5p in the LN metastasis of GC.

To further elucidate the target mRNAs of miR-204-5p regulation that contributed to the suppressive role of GC metastasis, complementary sequence of miR-204-5p is identified in the 3'-UTR of Chemokine ligand 12 (CXCL12)/chemokine receptor 4 (CXCR4) mRNA by Targetscan. Previous studies have shown the CXCL12/CXCR4 axis is positively associated with the aggressive phenotypes of GC 27-30. Further study revealed that CXCL12 and CXCR4 protein expression is increased in GC tissue with lymph nodes (LN) metastatic and mainly expressed in metastatic lymph nodes 31,32. CXCR4 is essential for metastatic spread to organs where CXCL12 is expressed 33,34. Compared with tissue without CXCR4 expression, CXCL12 attracts the organ-specific metastasis of CXCR4 expressing tumors, lead to more aggressive behaviors. And blockage of CXCL12 and CXCR4 could inhibit GC cells growth and invasion 35. In our current study, miR-204-5p was found as a regulator of CXCL12 and CXCR4 expression. Enforced expression of miR-204-5p could lead down-regulation of CXCR4 and CXCL12 expression in both mRNA and protein levels in GC cells. These results suggested that miR-204-5p involved in the post-transcriptional regulation of CXCR4 and CXCL12 expression in GC.

Next, we investigated the biological function of miR-204-5p in GC cells. Restoring miR-204-5p strongly inhibited the migration and invasion of GC cells. These data further confirmed that the low expression of miR-204-5p critically contributed to the metastasis of GC.

Conclusion, our clinical and experimental evidence strongly support that miR-204-5p act as a tumor suppressor in GC by targeting CXCR4 and CXCL12, thus regulating invasion and migration. Low levels of miR-204-5p significantly associated with LN metastasis of GC. Thus, miR-204-5p may be a potential molecular target to control metastatic GC.

## Figures and Tables

**Table 1:** Data of the patients in this study

Variables	GC (n=86)	benign patients^a^ (n=72)	p value
Age (mean, years)	61.44	59.21	0.126
Gender (male/female)	72/14	52/20	0.085
T stage (T1&2/T3&4)	61/25		
lymph metastasis (Y/N)	35/51		
Distant metastasis (Y/N)	12/74		
Clinical stage (I&II/III&IV)	48/38		
Lauren classification (diffuse/intestinal/mixed)	38/20/28		

a: including 40 cases of colorectal polyps, 2 cases of gastroesophageal reflux disease and 30 cases of gastric polyps.

**Table 2:** Relationship between miR-204-5p expression and clinicopathological characteristics of GC patients

Variables	Tissue interquartile				Serum interquartile			
	N	Median	Range	p	N	Median	Range	p
**Age**								
≤60	19				38	1.24	1.27	
>60	25				48	1.37	1.19	
**Gender**				0.630				0.13
Male	37	0.77	1.1		72	1.41	1.29	
Female	7	1.17	0.27		14	1.13	0.31	
**Lauren classification**				0.098				0.4
Diffuse	19	0.6	0.88		38	1.25	1.11	
Intestinal	15	1.33	1.3		28	1.49	1.04	
Mixed	10	0.64	0.94		20	1.13	1.66	
**T stage**				0.183				0.845
T1	7	1.02	0.93		14	1.17	1.17	
T2	6	1.1	0.95		11	1.33	0.85	
T3	5	1.86	2.64		10	1.3	1.35	
T4	26	0.49	1		51	1.36	1.2	
**N stage**				0.001				0.022
N0	24	1.26	0.76		51	1.43	1.58	
N1	5	0.33	0.86		12	2.05	1.51	
N2	10	0.41	0.27		14	1.15	0.27	
N3	5	0.22	0.57		9	0.68	0.85	
**M stage**				0.289				0.27
M0	38	0.98	1.17		74	1.3	1.12	
M1	6	0.41	0.94		12	1.54	4.2	
**Clinical stage**				0.024				0.322
I	10	1.1	0.62		20	1.34	1.08	
II	11	1.26	1.06		15	1.47	2.17	
III	17	0.32	1.02		39	1.24	1.31	
IV	6	0.41	0.94		12	1.54	4.2	

## References

[1] Kong F, Sun T, Kong X, Xie D, Li Z, Xie K (2018). Kruppel-like Factor 4 Suppresses Serine/Threonine Kinase 33 Activation and Metastasis of Gastric Cancer through Reversing Epithelial-Mesenchymal Transition. CLIN CANCER RES/8738.

[2] Van Cutsem E, Sagaert X, Topal B, Haustermans K, Prenen H (2016). Gastric cancer. Lancet (London, England).

[3] Badgwell B, Blum M, Estrella J, Ajani J (2016). Personalised therapy for localised gastric and gastro-oesophageal adenocarcinoma. LANCET ONCOL/26509.

[4] Cheng Y, Qu J, Che X, Xu L, Song N, Ma Y (2017). CXCL12/SDF-1alpha induces migration via SRC-mediated CXCR4-EGFR cross-talk in gastric cancer cells. ONCOL LETT/1482.

[5] Jiang YX, Yang SW, Li PA, Luo X, Li ZY, Hao YX (2017). The promotion of the transformation of quiescent gastric cancer stem cells by IL-17 and the underlying mechanisms. ONCOGENE/7932.

[6] Yang S, Sheng N, Pan L, Cao J, Liu J, Ma R (2018). microRNA-3129 promotes cell proliferation in gastric cancer cell line SGC7901 via positive regulation of pRb. BRAZ J MED BIOL RES/1146.

[7] Wu ZH, Lin C, Liu CC, Jiang WW, Huang MZ, Liu X (2018). MiR-616-3p promotes angiogenesis and EMT in gastric cancer via the PTEN/AKT/mTOR pathway. Biochem Biophys Res Commun.

[8] Wu N, Han Y, Liu H, Jiang M, Chu Y, Cao J (2018). miR-5590-3p inhibited tumor growth in gastric cancer by targeting DDX5/AKT/m-TOR pathway. Biochem Biophys Res Commun.

[9] Li W, Gao YQ (2018). MiR-217 is involved in the carcinogenesis of gastric cancer by down-regulating CDH1 expression. KAOHSIUNG J MED SCI/1.

[10] Zhang F, Li K, Pan M, Li W, Wu J, Li M (2018). miR-589 promotes gastric cancer aggressiveness by a LIFR-PI3K/AKT-c-Jun regulatory feedback loop. Journal of experimental & clinical cancer research: CR.

[11] Qi R, Wang DT, Xing LF, Wu ZJ (2018). miRNA-21 promotes gastric cancer growth by adjusting prostaglandin E2. Eur Rev Med Pharmacol Sci.

[12] Chang H, Kim N, Park JH, Nam RH, Choi YJ, Lee HS (2015). Different microRNA expression levels in gastric cancer depending on Helicobacter pylori infection. Gut and liver.

[13] Juzenas S, Salteniene V, Kupcinskas J, Link A, Kiudelis G, Jonaitis L (2015). Analysis of Deregulated microRNAs and Their Target Genes in Gastric Cancer. PloS one.

[14] Zhang T, Liu C, Huang S, Ma Y, Fang J, Chen Y (2017). A Downmodulated MicroRNA Profiling in Patients with Gastric Cancer. Gastroenterology research and practice.

[15] Zhang L, Wang X, Chen P (2013). MiR-204 down regulates SIRT1 and reverts SIRT1-induced epithelial-mesenchymal transition, anoikis resistance and invasion in gastric cancer cells. BMC cancer.

[16] Sacconi A, Biagioni F, Canu V, Mori F, Di Benedetto A, Lorenzon L (2012). miR-204 targets Bcl-2 expression and enhances responsiveness of gastric cancer. Cell death & disease.

[17] Zhang B, Yin Y, Hu Y, Zhang J, Bian Z, Song M (2015). MicroRNA-204-5p inhibits gastric cancer cell proliferation by downregulating USP47 and RAB22A. Medical oncology (Northwood, London, England).

[18] Liu J, Gao J, Du Y, Li Z, Ren Y, Gu J (2012). Combination of plasma microRNAs with serum CA19-9 for early detection of pancreatic cancer. Int J Cancer.

[19] Li W, Ng JM, Wong CC, Ng EKW, Yu J (2018). Molecular alterations of cancer cell and tumour microenvironment in metastatic gastric cancer. ONCOGENE.

[20] Marchet A, Mocellin S, Ambrosi A, Morgagni P, Garcea D, Marrelli D (2007). The ratio between metastatic and examined lymph nodes (N ratio) is an independent prognostic factor in gastric cancer regardless of the type of lymphadenectomy: results from an Italian multicentric study in 1853 patients. ANN SURG/8569.

[21] Arigami T, Natsugoe S, Uenosono Y, Yanagita S, Arima H, Hirata M (2009). CCR7 and CXCR4 expression predicts lymph node status including micrometastasis in gastric cancer. INT J ONCOL/3018.

[22] Li M, Shen Y, Wang Q, Zhou X (2019). MiR-204-5p promotes apoptosis and inhibits migration of osteosarcoma via targeting EBF2. Biochimie.

[23] Hong BS, Ryu HS, Kim N, Kim J, Lee E, Moon H (2019). Tumor Suppressor miRNA-204-5p Regulates Growth, Metastasis, and Immune Microenvironment Remodeling in Breast Cancer. Cancer research.

[24] Palkina N, Komina A, Aksenenko M, Moshev A, Savchenko A, Ruksha T (2018). miR-204-5p and miR-3065-5p exert antitumor effects on melanoma cells. Oncology letters.

[25] Lin YC, Lin JF, Tsai TF, Chou KY, Chen HE, Hwang TI (2017). Tumor suppressor miRNA-204-5p promotes apoptosis by targeting BCL2 in prostate cancer cells. Asian journal of surgery.

[26] Bian Z, Jin L, Zhang J, Yin Y, Quan C, Hu Y (2016). LncRNA-UCA1 enhances cell proliferation and 5-fluorouracil resistance in colorectal cancer by inhibiting miR-204-5p. Scientific reports.

[27] Satomura H, Sasaki K, Nakajima M, Yamaguchi S, Onodera S, Otsuka K (2014). Can expression of CXCL12 and CXCR4 be used to predict survival of gastric cancer patients?. ANTICANCER RES/1895.

[28] Zheng S, Shi L, Zhang Y, He T (2014). Expression of SNCG, MAP2, SDF-1 and CXCR4 in gastric adenocarcinoma and their clinical significance. Int J Clin Exp Pathol.

[29] Izumi D, Ishimoto T, Miyake K, Sugihara H, Eto K, Sawayama H (2016). CXCL12/CXCR4 activation by cancer-associated fibroblasts promotes integrin beta1 clustering and invasiveness in gastric cancer. INT J CANCER/5531.

[30] Cheng Y, Song Y, Qu J, Che X, Song N, Fan Y (2018). The Chemokine Receptor CXCR4 and c-MET Cooperatively Promote Epithelial-Mesenchymal Transition in Gastric Cancer Cells. TRANSL ONCOL/3077.

[31] Ying J, Xu Q, Zhang G, Liu B, Zhu L (2012). The expression of CXCL12 and CXCR4 in gastric cancer and their correlation to lymph node metastasis. Medical oncology (Northwood, London, England).

[32] Iwasa S, Yanagawa T, Fan J, Katoh R (2009). Expression of CXCR4 and its ligand SDF-1 in intestinal-type gastric cancer is associated with lymph node and liver metastasis. ANTICANCER RES/1895.

[33] Barbieri F, Bajetto A, Stumm R, Pattarozzi A, Porcile C, Zona G (2008). Overexpression of stromal cell-derived factor 1 and its receptor CXCR4 induces autocrine/paracrine cell proliferation in human pituitary adenomas. CLIN CANCER RES/8738.

[34] Burger JA, Kipps TJ (2006). CXCR4: a key receptor in the crosstalk between tumor cells and their microenvironment. BLOOD/11841.

[35] Lin XL, Xu Q, Tang L, Sun L, Han T, Wang LW (2017). Regorafenib inhibited gastric cancer cells growth and invasion via CXCR4 activated Wnt pathway. PloS one.

